# Supplementary material for: Computational Tool to Design Small Synthetic Inhibitors Selective for XIAP-BIR3 Domain
Source: Molecules. 2023 Jun 30;28(13):5155. doi: 10.3390/molecules28135155 (PMC10343376; doi:10.3390/molecules28135155)
Supplement: Supplementary file 1 [file molecules-28-05155-s001.zip › molecules-2456939-supplementary.pdf]

Supporting Information

Computational tool to design non peptidomimetic antagonists selectives for XIAP BIR3 domain

Marc Farag [1], Charline Kieffer [1], Nicolas Guedeney [1]Anne Sophie Voisin-Chiret [1] and Jana Sopkova-de Oliveira Santos [1,*]

[1] Normandie Univ, Unicaen, Cermn, 14000 Caen, France; marc.ragui@unicaen.fr (M.F.); anne-sophie.voisin@unicaen.fr (AVC.); charline.kieffer@unicaen.fr (C.K.); jana.sopkova@unicaen.fr (J.S.)

* Correspondence: jana.sopkova@unicaen.fr; Tel.: +33231566821

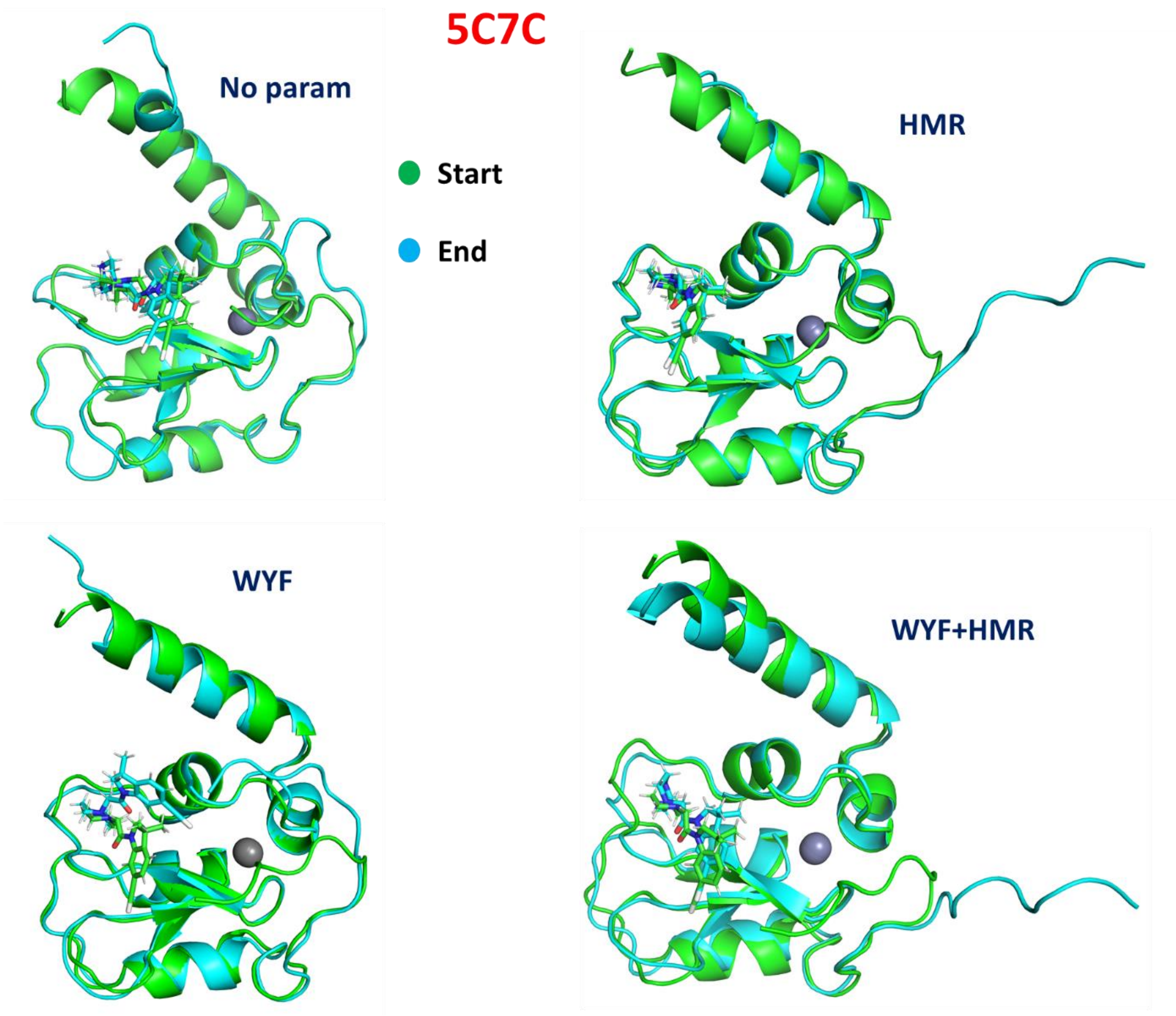

5C7C
No param
Start
End
HMR
WYF
WYF+HMR

5OQW

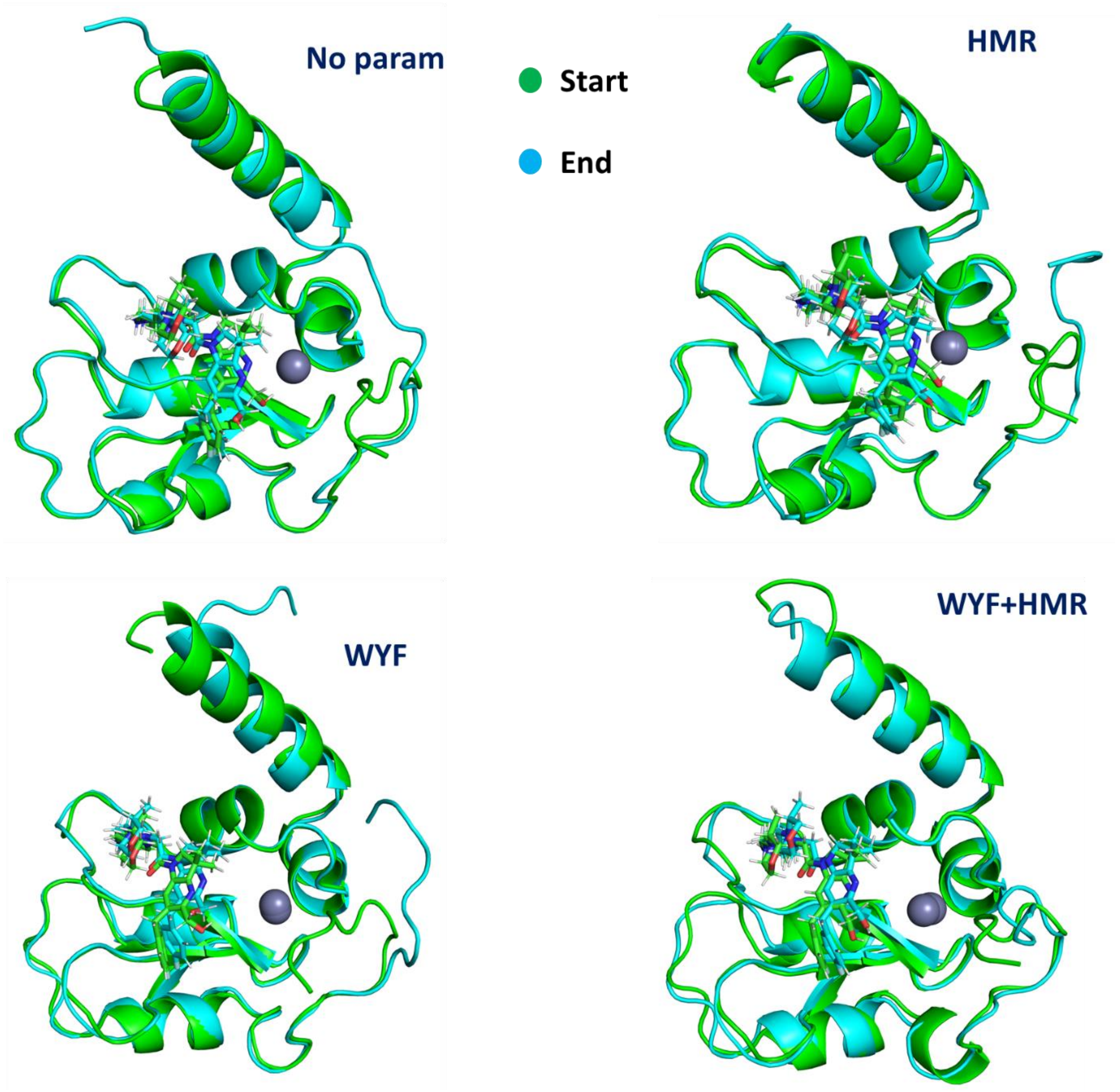

5M6M

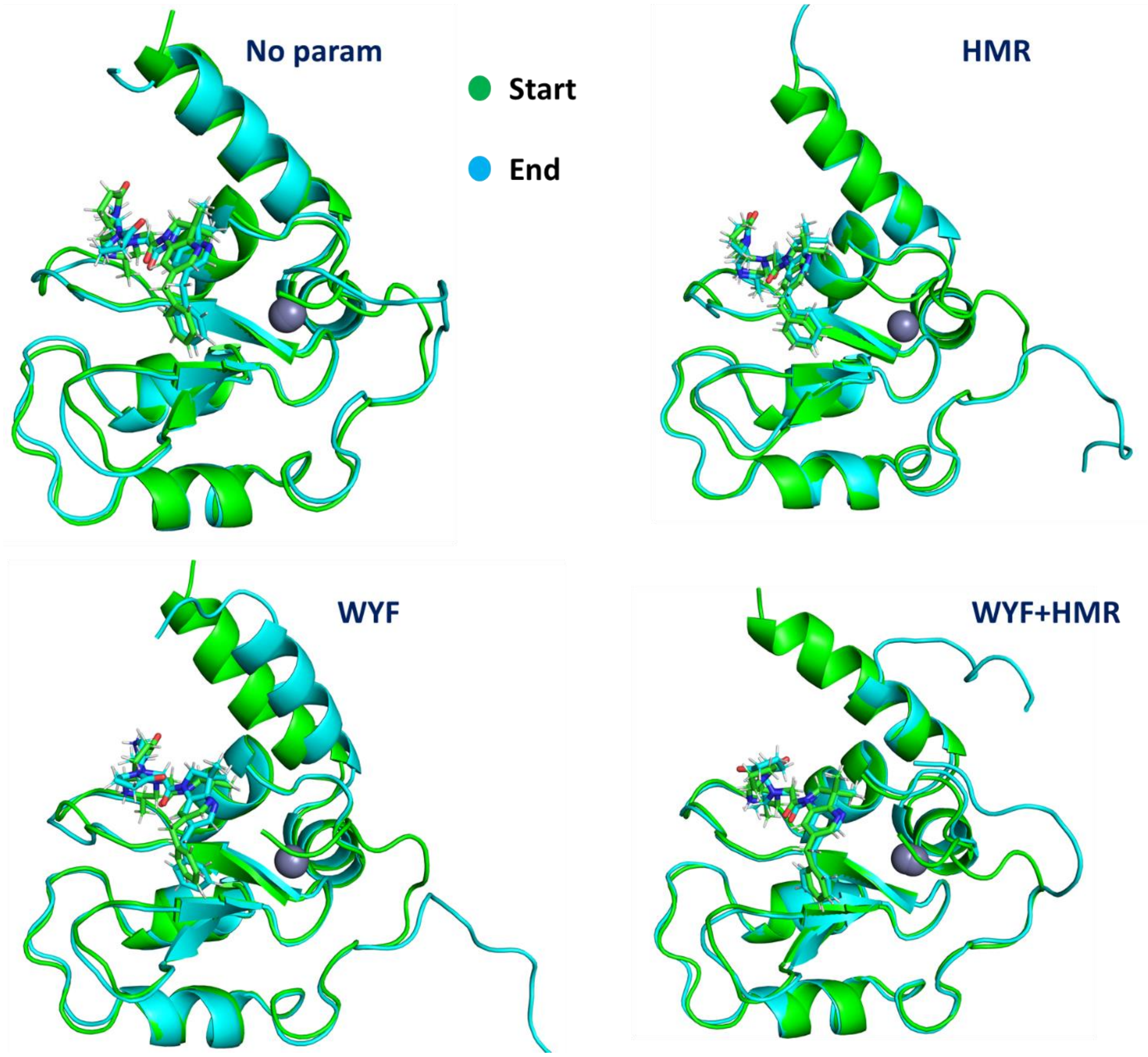

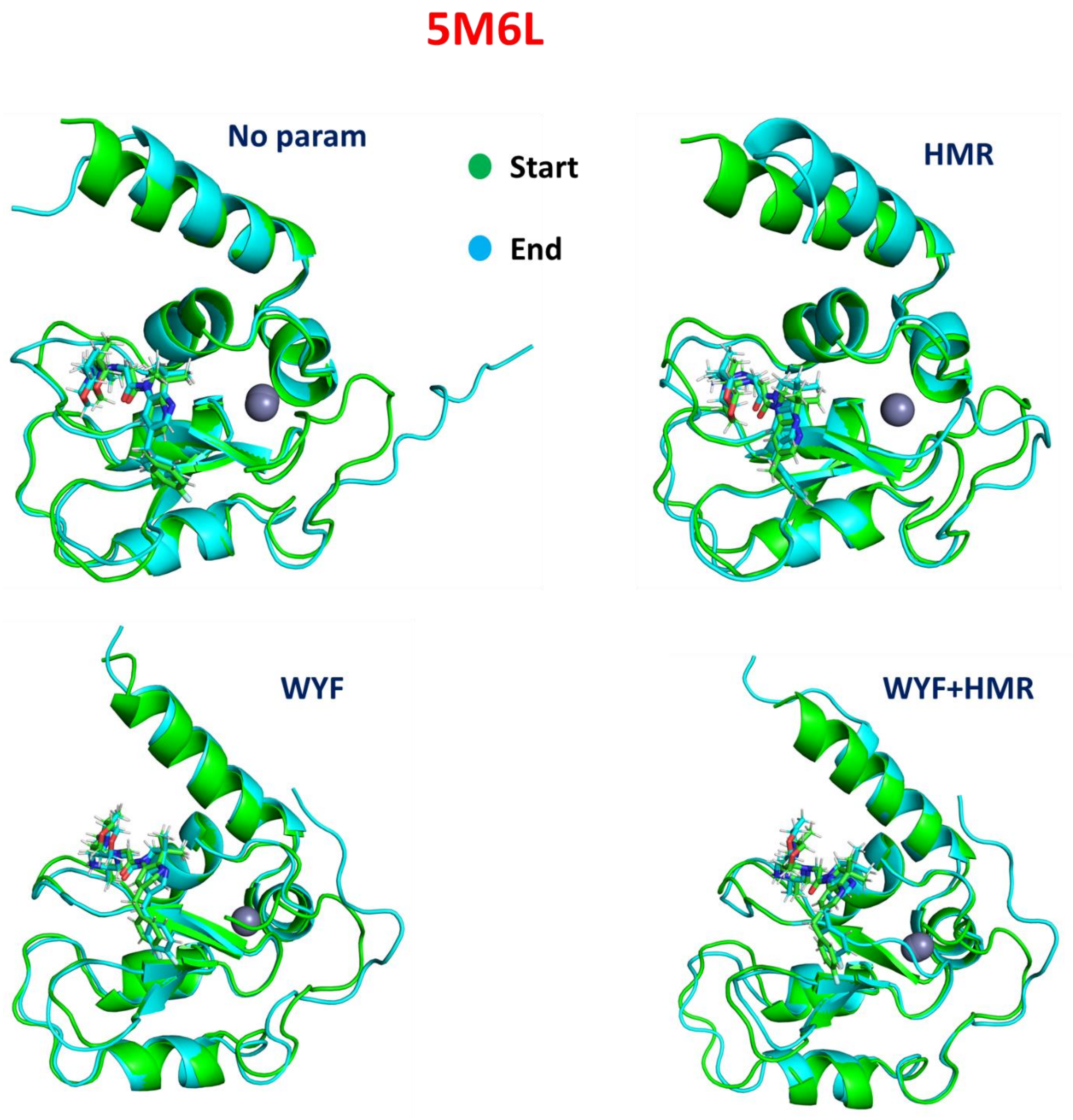

Figure S1: The alignment of the end structure from molecular dynamics simulation on the initial one for four studied complexes with synthetic ligands.

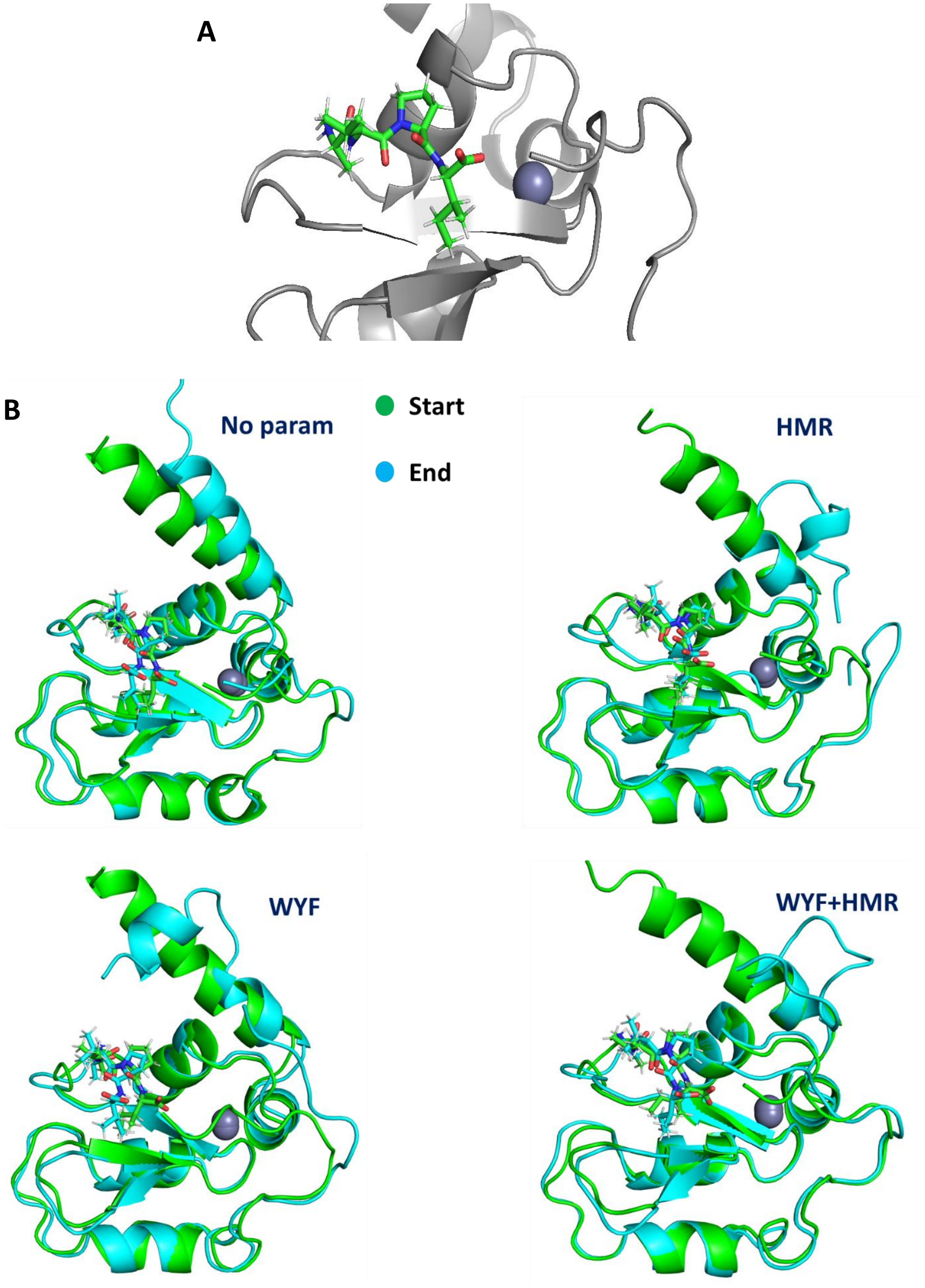

Figure S2: (A) Selected pose from docking for AVPI into 5C7C structure (B)The alignment of the end structure from molecular dynamics simulation on the initial one for four studied complexes with synthetic ligands.

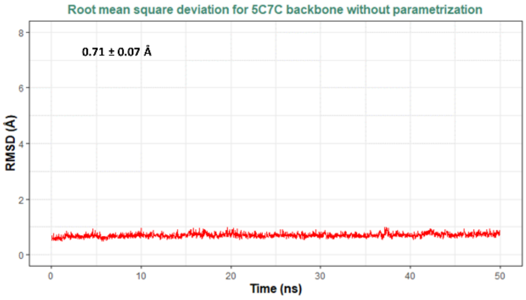
Root mean square deviation for 5C7C backbone without parametrization
0.71 ± 0.07 Å
RMSD (Å)
Time (ns)

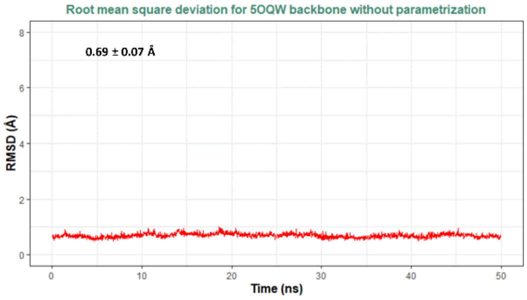
Root mean square deviation for 5OQW backbone without parametrization
0.69 ± 0.07 Å
RMSD (Å)
Time (ns)

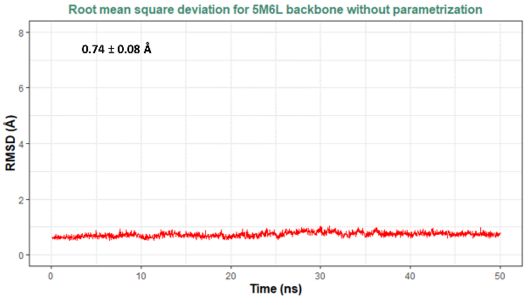
Root mean square deviation for 5M6L backbone without parametrization
0.74 ± 0.08 Å
RMSD (Å)
Time (ns)

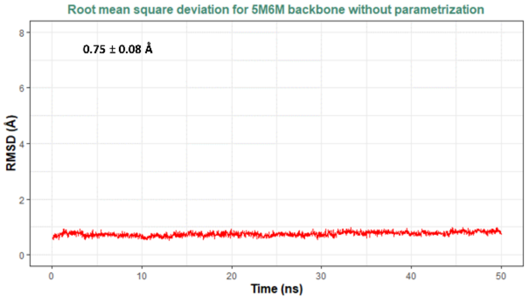
Root mean square deviation for 5M6M backbone without parametrization
0.75 ± 0.08 Å
RMSD (Å)
Time (ns)

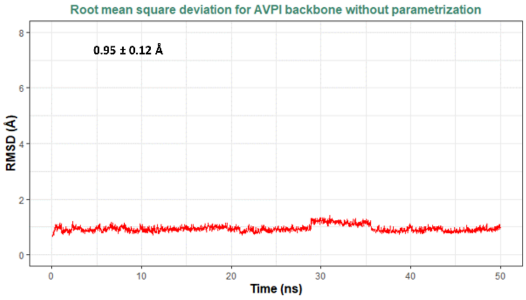
Root mean square deviation for AVPI backbone without parametrization
0.95 ± 0.12 Å
RMSD (Å)
Time (ns)

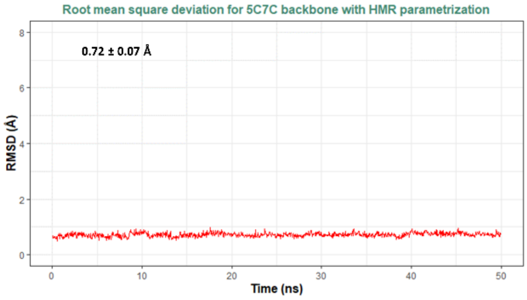
Root mean square deviation for 5C7C backbone with HMR parametrization
0.72 ± 0.07 Å
RMSD (Å)
Time (ns)

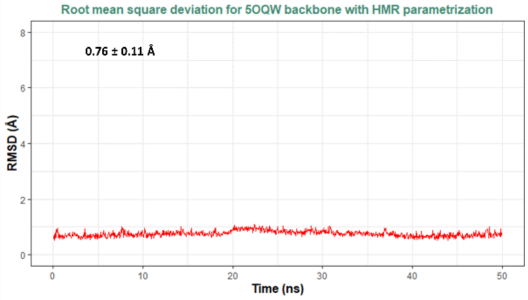
Root mean square deviation for 5OQW backbone with HMR parametrization
0.76 ± 0.11 Å
RMSD (Å)
Time (ns)

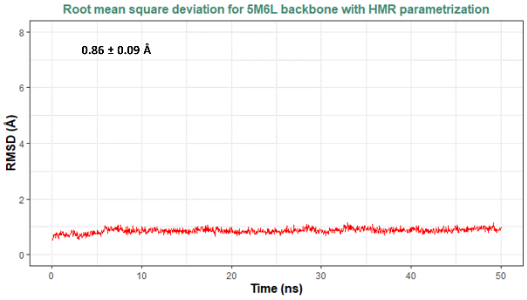
Root mean square deviation for 5M6L backbone with HMR parametrization
0.86 ± 0.09 Å
RMSD (Å)
Time (ns)

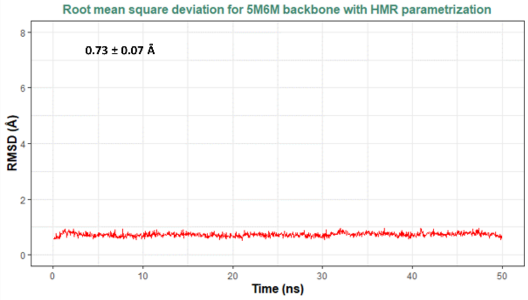
Root mean square deviation for 5M6M backbone with HMR parametrization
0.73 ± 0.07 Å
RMSD (Å)
Time (ns)

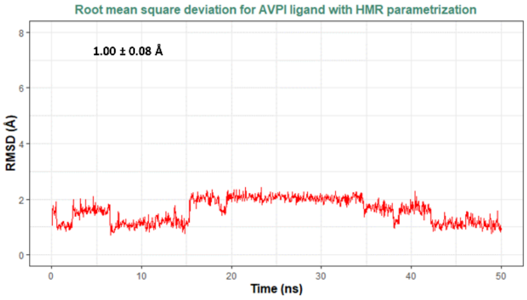
Root mean square deviation for AVPI ligand with HMR parametrization
1.00 ± 0.08 Å
RMSD (Å)
Time (ns)

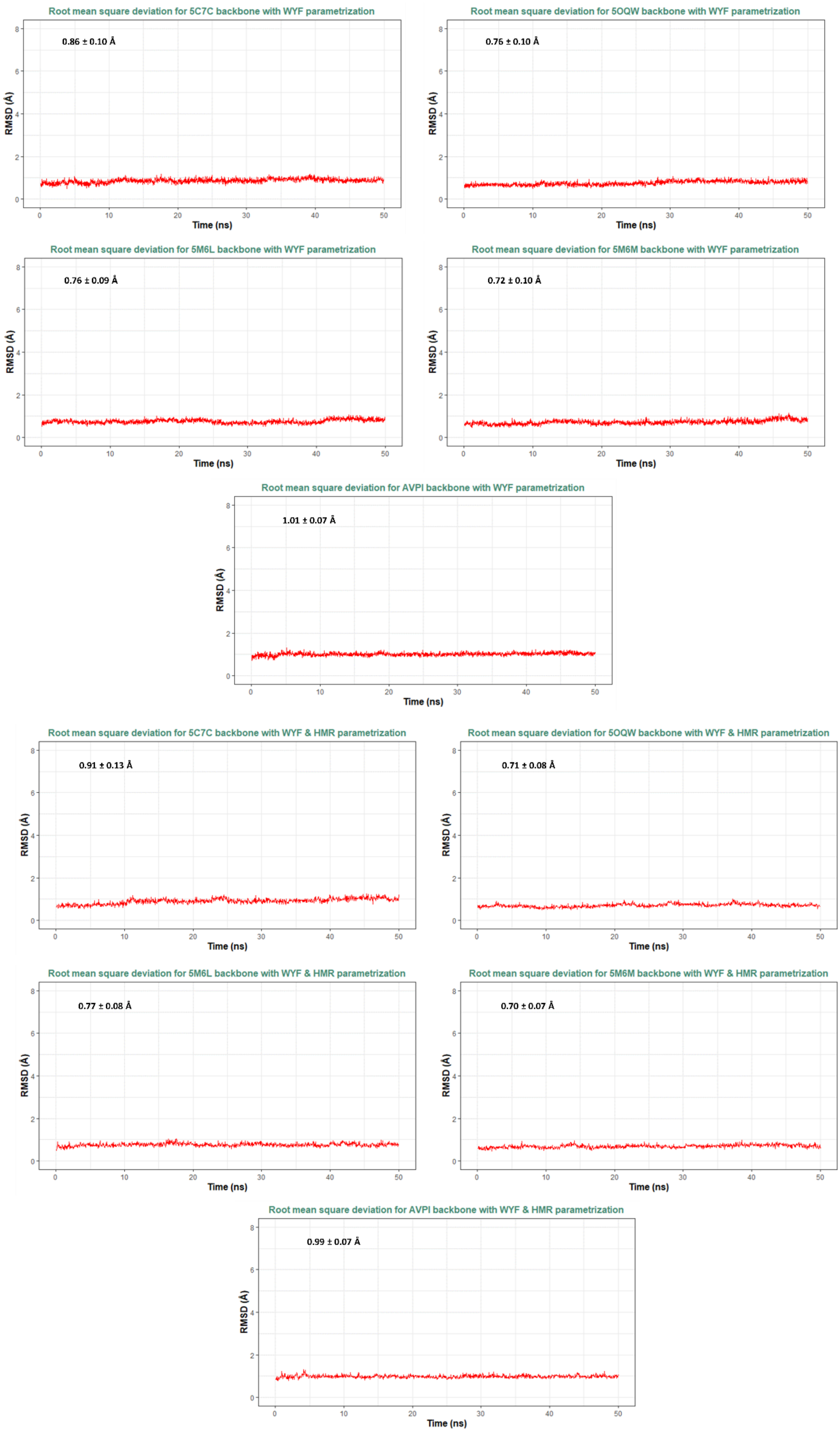

Figure S3: The RMSD of the protein backbone atoms along the MD simulations. The conformations were aligned excluding the C and N-terminal helices. The RMSDs were calculated only on the XIAP-BIR3 core (without N and C termini).

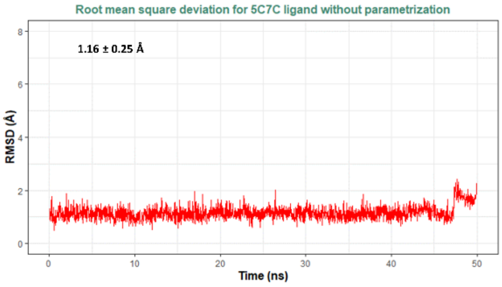
Root mean square deviation for 5C7C ligand without parametrization
1.16 ± 0.25 Å
RMSD (Å)
Time (ns)

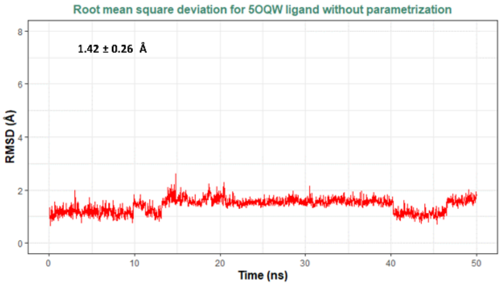
Root mean square deviation for 5OQW ligand without parametrization
1.42 ± 0.26 Å
RMSD (Å)
Time (ns)

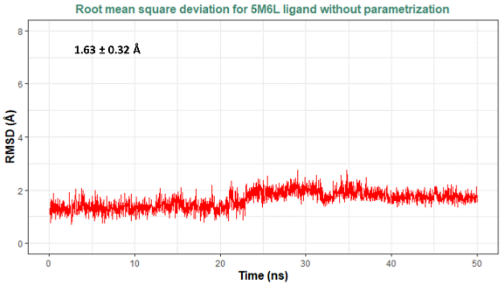
Root mean square deviation for 5M6L ligand without parametrization
1.63 ± 0.32 Å
RMSD (Å)
Time (ns)

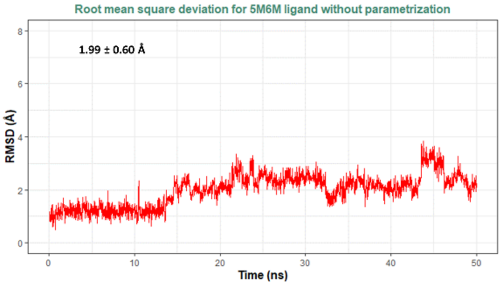
Root mean square deviation for 5M6M ligand without parametrization
1.99 ± 0.60 Å
RMSD (Å)
Time (ns)

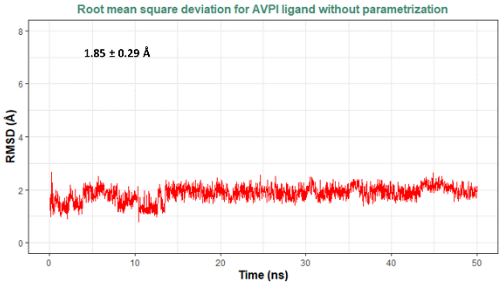
Root mean square deviation for AVPI ligand without parametrization
1.85 ± 0.29 Å
RMSD (Å)
Time (ns)

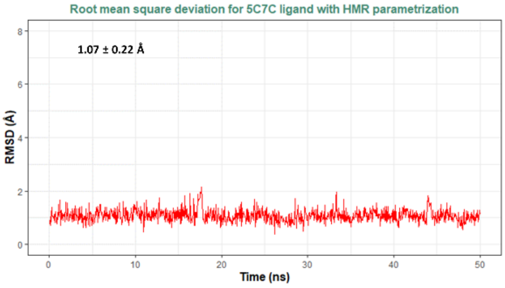
Root mean square deviation for 5C7C ligand with HMR parametrization
1.07 ± 0.22 Å
RMSD (Å)
Time (ns)

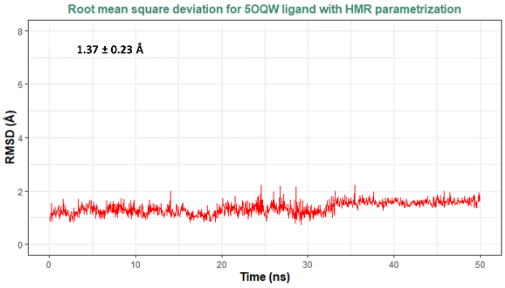
Root mean square deviation for 5OQW ligand with HMR parametrization
1.37 ± 0.23 Å
RMSD (Å)
Time (ns)

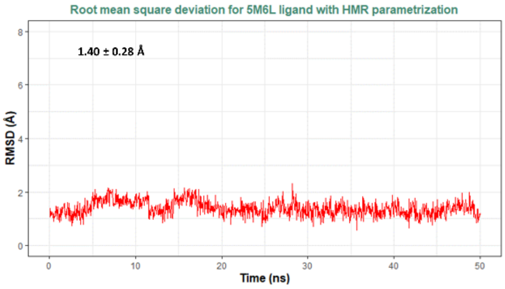
Root mean square deviation for 5M6L ligand with HMR parametrization
1.40 ± 0.28 Å
RMSD (Å)
Time (ns)

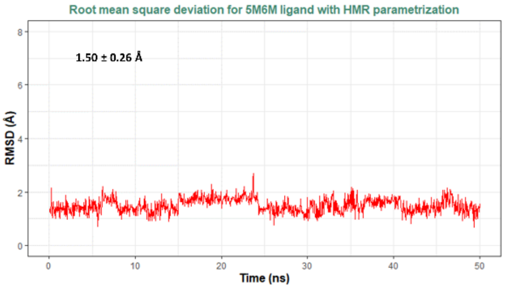
Root mean square deviation for 5M6M ligand with HMR parametrization
1.50 ± 0.26 Å
RMSD (Å)
Time (ns)

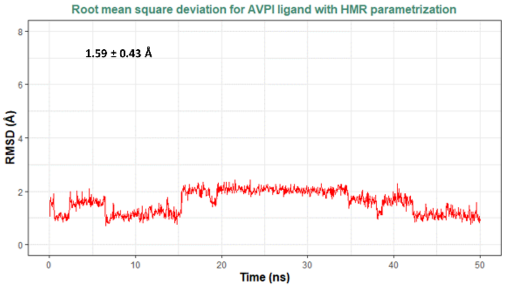
Root mean square deviation for AVPI ligand with HMR parametrization
1.59 ± 0.43 Å
RMSD (Å)
Time (ns)

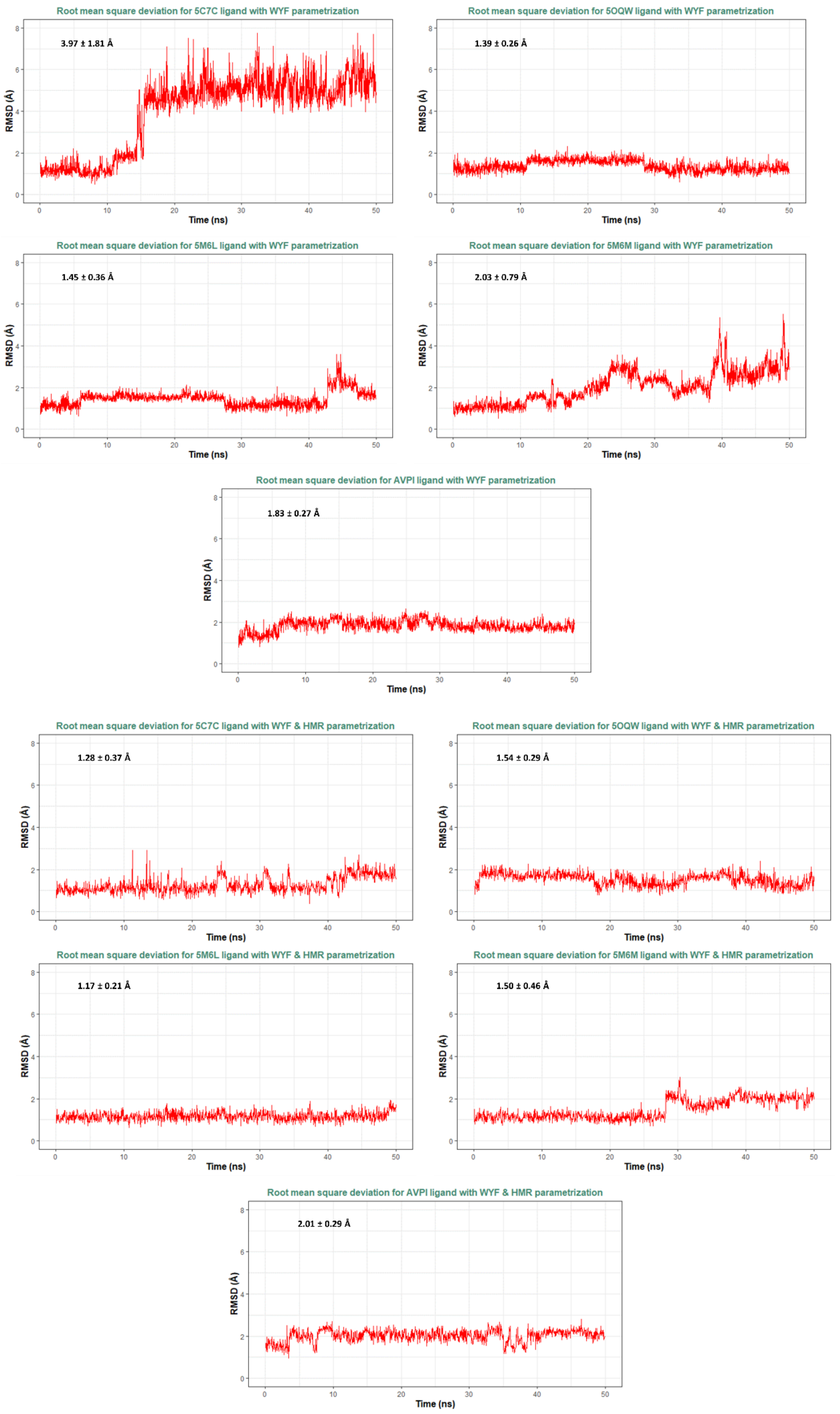

Figure S4: The RMSD of the ligands along the MD simulations. The complexes generated from trajectories were aligned using the protein backbone atoms excluding the C and N-terminal helices.

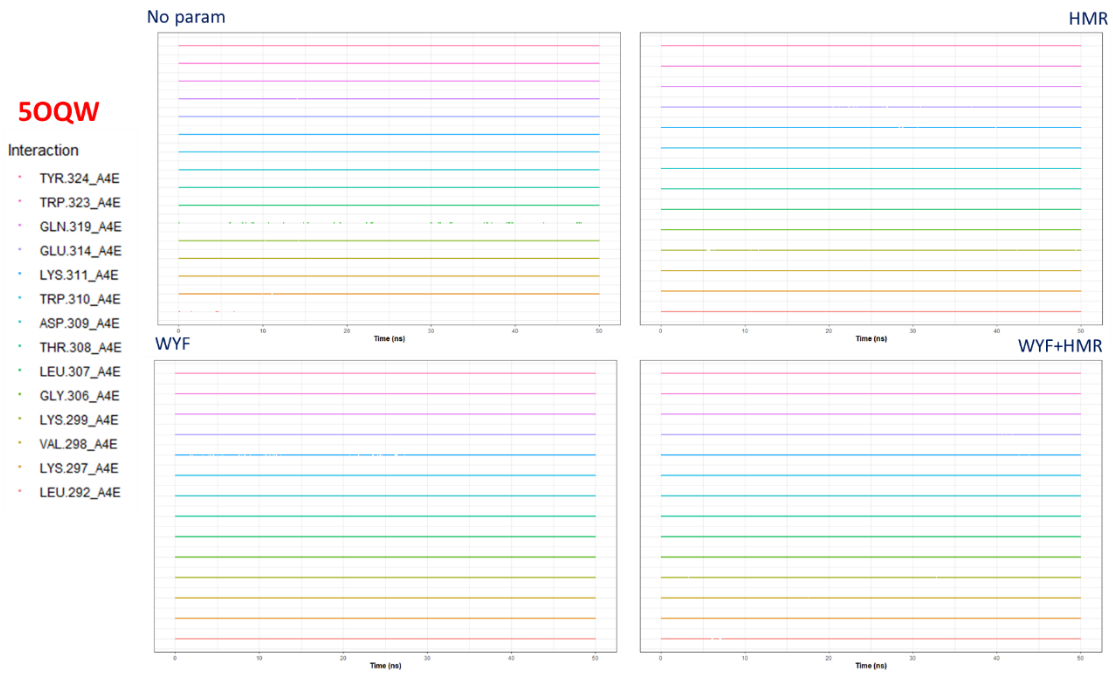
5OQW
No param
HMR
WYF
WYF+HMR
Interaction
TYR.324_A4E
TRP.323_A4E
GLN.319_A4E
GLU.314_A4E
LYS.311_A4E
TRP.310_A4E
ASP.309_A4E
THR.308_A4E
LEU.307_A4E
GLY.306_A4E
LYS.299_A4E
VAL.298_A4E
LYS.297_A4E
LEU.292_A4E
Time (ns)

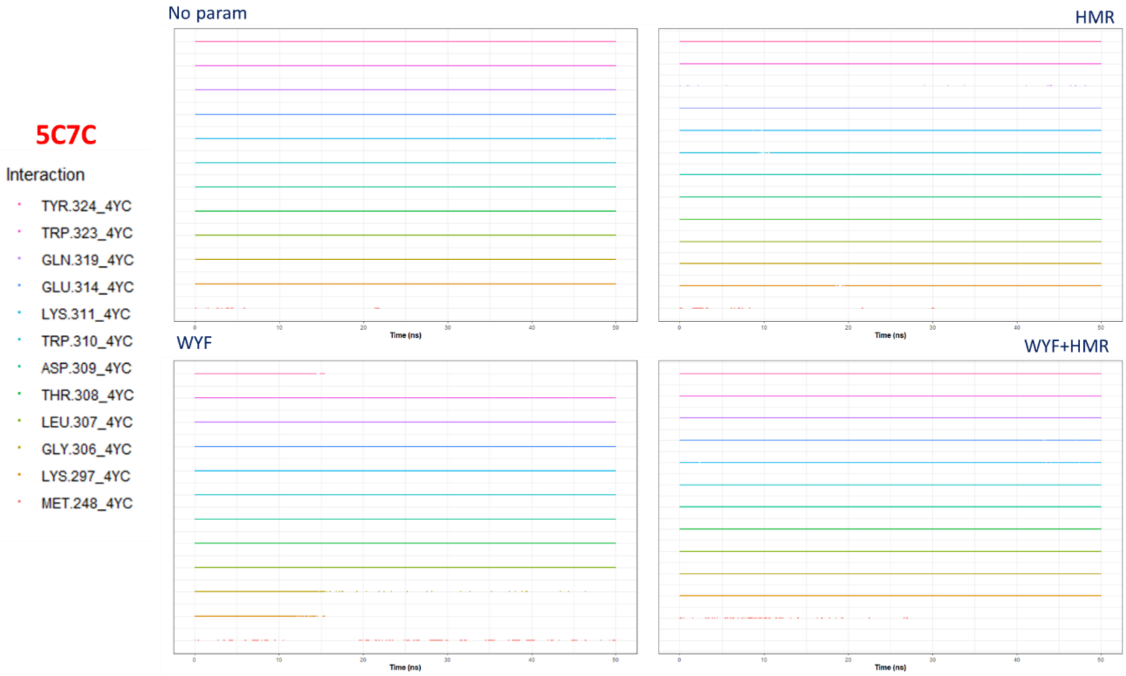
5C7C
No param
HMR
WYF
WYF+HMR
Interaction
TYR.324_4YC
TRP.323_4YC
GLN.319_4YC
GLU.314_4YC
LYS.311_4YC
TRP.310_4YC
ASP.309_4YC
THR.308_4YC
LEU.307_4YC
GLY.306_4YC
LYS.297_4YC
MET.248_4YC
Time (ns)

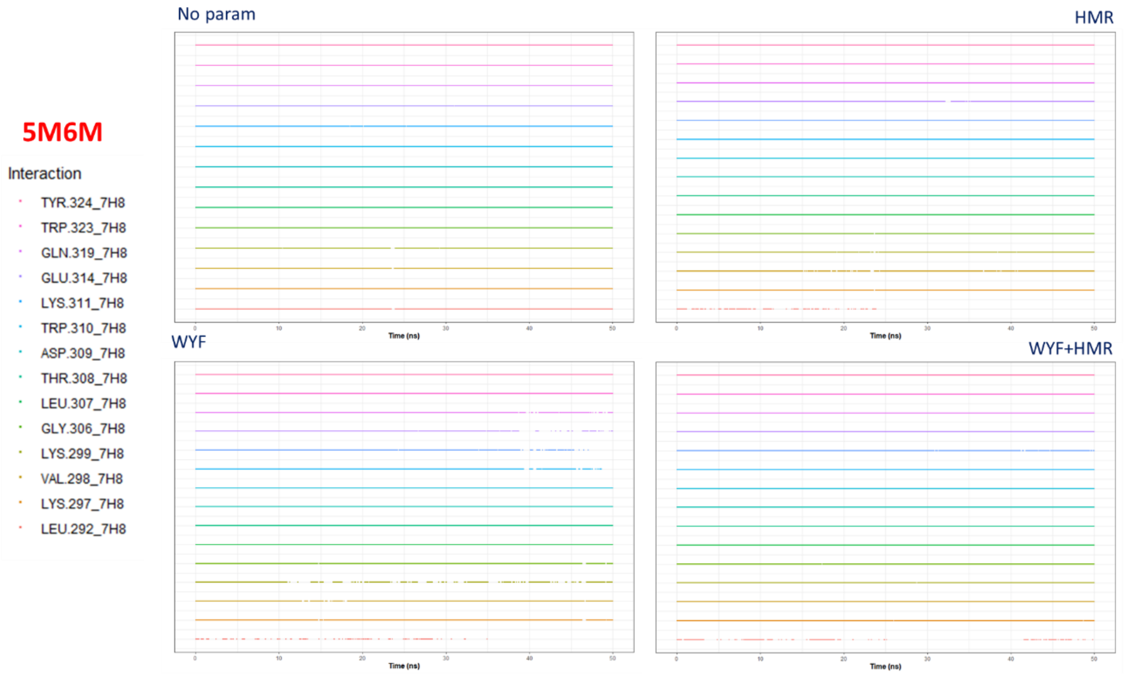
5M6M
Interaction
TYR.324_7H8
TRP.323_7H8
GLN.319_7H8
GLU.314_7H8
LYS.311_7H8
TRP.310_7H8
ASP.309_7H8
THR.308_7H8
LEU.307_7H8
GLY.306_7H8
LYS.299_7H8
VAL.298_7H8
LYS.297_7H8
LEU.292_7H8
No param
HMR
WYF
WYF+HMR
Time (ns)

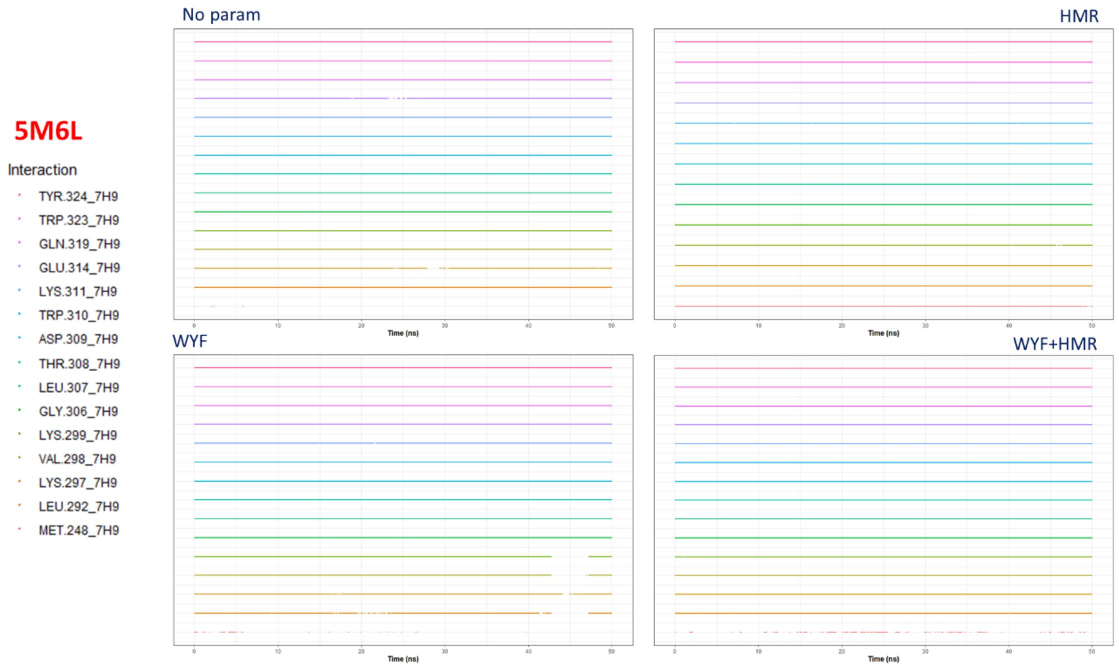
5M6L
Interaction
TYR.324_7H9
TRP.323_7H9
GLN.319_7H9
GLU.314_7H9
LYS.311_7H9
TRP.310_7H9
ASP.309_7H9
THR.308_7H9
LEU.307_7H9
GLY.306_7H9
LYS.299_7H9
VAL.298_7H9
LYS.297_7H9
LEU.292_7H9
MET.248_7H9
No param
HMR
WYF
WYF+HMR
Time (ns)

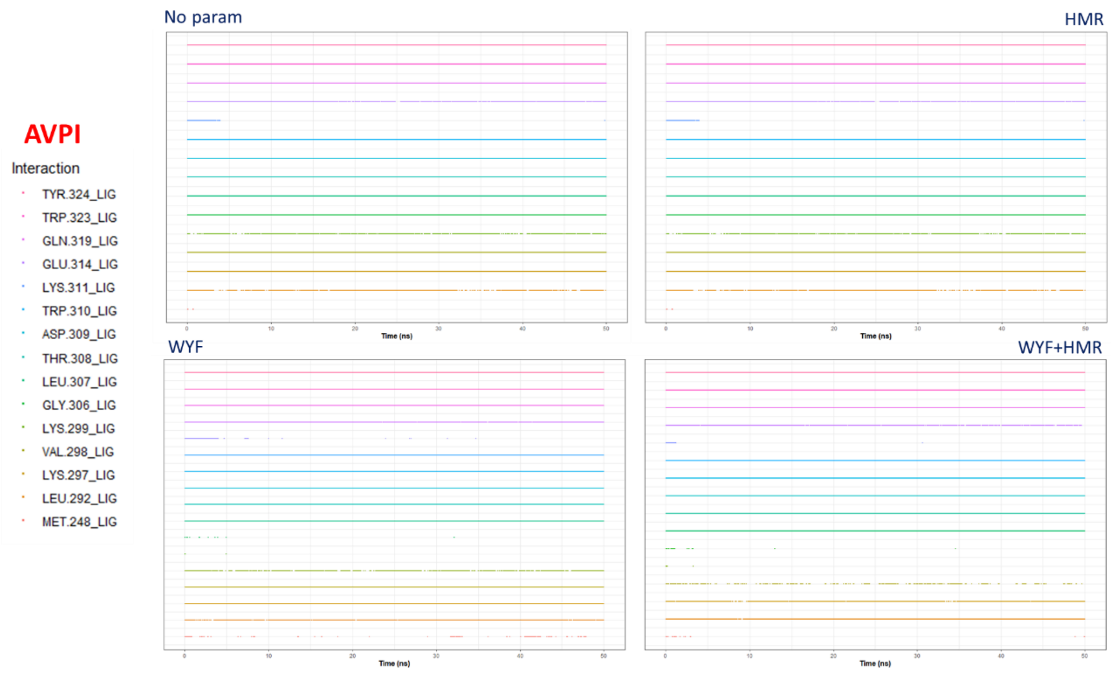

Figure S5: Key interacting residues of XIAP-BIR3 with ligands and their dynamical evolution along MD trajectories for all simulated complexes.

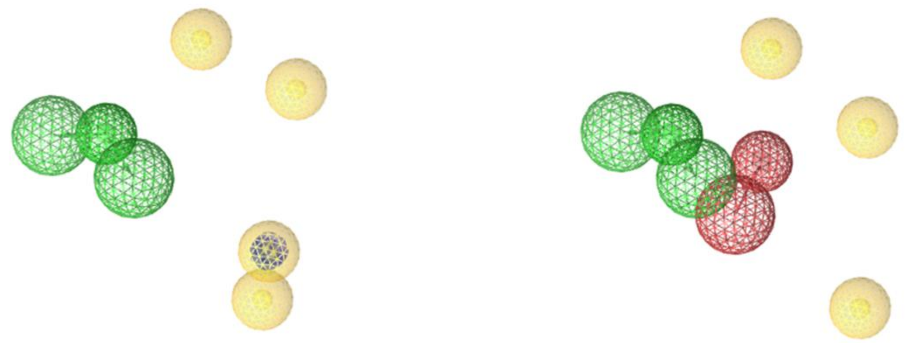

Pharmacophore from X-ray structure: 5M6L

Pharmacophore from 2500 frames of MD simulation (HMR additional parametrisation).

Figure S6: Two pharmacophores used to create the final pharmacophore. On the left, the one generated from 5M6L complex X-ray structure. On the right, the one generated from the 2500 frames of molecular dynamics simulations.

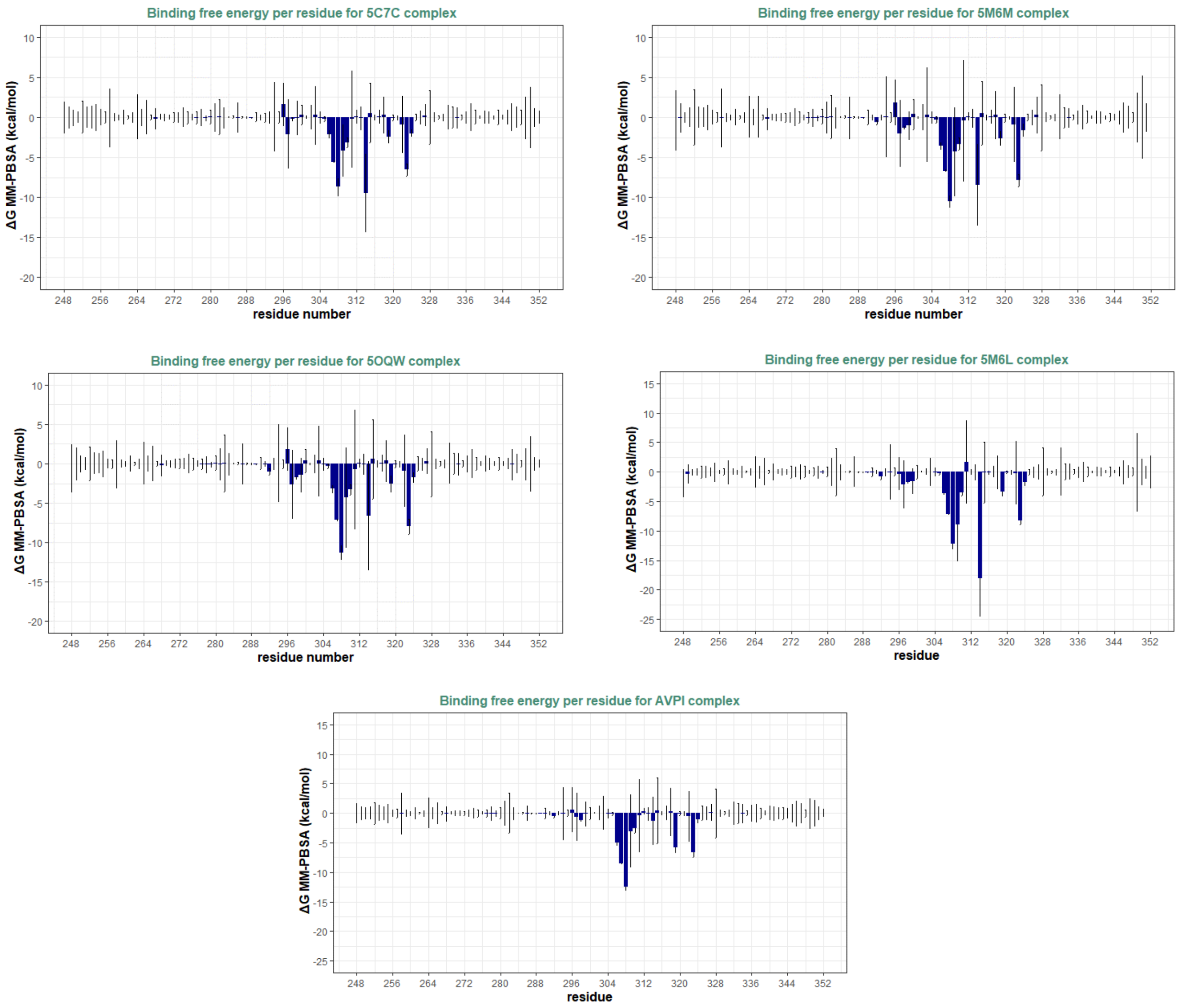

Figure S7: $\Delta G_{MM\text{-}PBSA}$ per residue without entropic term for the five studied complexes.

Table S1: Experimental binding free energy ΔG_{exp} and calculated ones using the MM-PBSA method $\Delta G_{MM-PBSA}$ for different parametrisations of the force field.

a) without entropic term

10 ns

	ΔG_{exp} [kcal/mol]	$\Delta G_{MM-PBSA}$ [kcal/mol]			
		Without additional parametrisation	HMR	WYF	HMR + WYF
5C7C	-7.29	-36.8 ± 3.0	-37.5 ± 4.7	-37.4 ± 3.5	-35.3 ± 3.8
5M6M	-10.20	-44.4 ± 4.3	-44.8 ± 3.6	-44.9 ± 3.8	-44.0 ± 4.2
5OQW	-10.26	-48.6 ± 4.0	-46.3 ± 4.0	-47.2 ± 3.7	-47.3 ± 3.9
5M6L	-11.49	-44.9 ± 4.0	-44.8 ± 4.2	-47.3 ± 3.6	-47.0 ± 3.0
AVPI	-8.91	-46.9 ± 5.3	-44.0 ± 7.9	-48.0 ± 5.2	-40.0 ± 8.3

25 ns

	ΔG_{exp} [kcal/mol]	$\Delta G_{MM-PBSA}$ [kcal/mol]			
		Without additional parametrisation	HMR	WYF	HMR + WYF
5C7C	-7.29	-36.9 ± 3.2	-36.3 ± 4.5	-32.7 ± 5.8	-34.8 ± 3.9
5M6M	-10.20	-38.4 ± 8.0	-43.0 ± 4.0	-42.3 ± 5.4	-43.2 ± 3.8
5OQW	-10.26	-44.8 ± 5.2	-44.4 ± 5.4	-47.3 ± 4.0	-46.0 ± 4.0
5M6L	-11.49	-44.4 ± 4.4	-44.1 ± 4.3	-45.6 ± 4.8	-46.8 ± 3.9
AVPI	-8.91	-45.6 ± 4.9	-41.6 ± 6.8	-45.9 ± 5.4	-38.4 ± 6.6

b) with entropic term calculated using normal mode analysis of harmonic frequencies (NM) or using the interaction entropy method (IE)

10 ns

	ΔG_{exp} [kcal/mol]	$\Delta G_{MM\text{-}PBSA}$ [kcal/mol]							
		Without additional parametrisation		HMR		WYF		HMR + WYF	
		NM	IE	NM	IE	NM	IE	NM	IE
5C7C	-7.29	-10.4 ± 3.1	-27.5 ± 3.0	-11.1 ± 4.9	-18.7 ± 5.2	-9.9 ± 3.6	-29.8 ± 3.5	-9.0 ± 3.9	-18.5 ± 4.0
5M6M	-10.20	-5.1 ± 4.4	- 33.5 ± 4.4	-1.3 ± 3.6	-36.2 ± 3.6	-3.9 ± 3.9	-31.3 ± 3.9	-2.3 ± 4.2	-33.5 ± 4.3
5OQW	-10.26	1.4 ± 4.0	-32.9 ± 4.1	3.0 ± 4.0	-37.9 ± 4.0	-3.6 ± 3.7	-39.1 ± 3.7	-3.9 ± 3.5	-39.0 ± 3.9
5M6L	-11.49	-3.6 ± 4.0	-26.1 ± 4.4	-1.1 ± 4.2	-26.7 ± 4.4	-5.1 ± 3.6	-40.4 ± 3.7	-6.2 ± 3.7	-38.5 ± 3.7
AVPI	-8.91	-4.3 ± 5.3	-33.6 ± 5.5	0.6 ± 7.9	-23.6 ± 8.6	-5.9 ± 5.2	-31.1 ± 5.7	7.0 ± 8.4	-16.8 ± 9.9

25 ns

	ΔG_{exp} [kcal/mol]	$\Delta G_{MM\text{-}PBSA}$ [kcal/mol]							
		Without additional parametrisation		HMR		WYF		HMR + WYF	
		NM	IE	NM	IE	NM	IE	NM	IE
5C7C	-7.29	-20.4 ± 3.6	-26.4 ± 3.2	-18.5 ± 5.0	-15.5 ± 4.9	-12.1 ± 5.0	-19.1 ± 5.9	-16.8 ± 4.4	-18.2 ± 4.1
5M6M	-10.20	-9.5 ± 8.2	- 7.81 ± 10.4	-18.2 ± 4.3	-34.0 ± 4.0	-14.0 ± 6.3	-15.2 ± 6.6	-8.2 ± 3.9	-33.0 ± 4.2
5OQW	-10.26	-16.7 ± 6.0	-27.5 ± 5.6	-13.9 ± 5.7	-23.7 ± 6.0	-19.4 ± 4.3	-34.4 ± 4.1	-19.1 ± 4.3	-33.7 ± 4.0
5M6L	-11.49	-18.0 ± 4.7	-21.8 ± 5.2	-17.7 ± 4.6	-26.5 ± 4.5	-20.2 ± 5.3	-29.6 ± 5.0	-22.4 ± 4.3	-25.2 ± 4.1
AVPI	-8.91	-19.4 ± 5.4	-33.3 ± 5.1	-12.7 ± 7.1	-22.7 ± 7.3	-19.0 ± 5.9	-26.1 ± 6.0	-9.8 ± 6.8	-19.6 ± 7.4
